# Boredom in Psychiatric Disorders: *Another Dimensional Construct?*

**DOI:** 10.1002/brb3.71718

**Published:** 2026-08-31

**Authors:** Riccardo Gurrieri, Matteo Gambini, Giorgia Sità, Elena Pescini, Federico Mucci, Donatella Marazziti

**Affiliations:** ^1^ Health Science Interdisciplinary Center School of Advanced Studies Pisa Italy; ^2^ Department of Clinical and Experimental Medicine, Section of Psychiatry University of Pisa Pisa Italy; ^3^ Department of Mental Health Lucca Zone, Azienda USL Toscana Nord Ovest Lucca Italy

**Keywords:** boredom, default mode network, psychiatry, psychopathology, transdiagnostic construct

## Abstract

**Purpose:**

Boredom is increasingly emerging as a clinically relevant construct in psychiatry, yet it remains insufficiently integrated into contemporary psychopathological models. Rather than reflecting a trivial or purely situational state, boredom may be conceptualized as an aversive condition arising when individuals desire meaningful engagement but are unable to sustain it. This review examines boredom across major psychiatric disorders, with particular attention to its neurophysiological, neurobiological, and clinical correlates.

**Methods:**

A narrative synthesis of conceptual, neurophysiological, neuroimaging, autonomic, and clinical literature on boredom was conducted, considering findings across major psychiatric disorders.

**Finding:**

Available evidence suggests that boredom is associated with alterations in attentional regulation, motivational processing, and internally oriented cognition. EEG studies report increased alpha activity, altered theta dynamics, and reduced indices of cognitive control, whereas functional neuroimaging findings highlight greater involvement of the default mode network and reduced engagement of fronto‐parietal control systems. Autonomic data further indicate that boredom is not a uniform low‐arousal state, but a dynamic psychophysiological condition characterized by fluctuating patterns of disengagement and re‐engagement. Clinically, boredom appears to cut across diagnostic categories, including attention‐deficit/hyperactivity disorder, mood and anxiety disorders, psychotic spectrum conditions, addictions, eating disorders, personality disorders, and suicidality. Across these conditions, it may function both as a vulnerability factor and as a mechanism contributing to maladaptive coping, impaired functioning, and poorer treatment adherence.

**Conclusion:**

Overall, the literature supports the view that boredom may represent a dimensional and transdiagnostic construct of potential relevance for psychiatric research and practice. A more systematic assessment of boredom may improve psychopathological characterization and help identify novel therapeutic targets.

## Graphical abstract

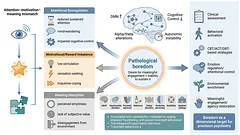

## 1 Introduction

Boredom is a complex and multidimensional human experience, defined as an aversive state in which an individual desires to engage in a meaningful activity but feels unable to do so (Bench and Lench 2013; Cannito et al. 2023; Chen and Rau 2022; Eastwood et al. 2012; Ndetei et al. 2023; Seiler and Dan 2024). It represents a negative emotional state characterized by dissatisfaction and difficulty sustaining attention, emerging from a discrepancy between available stimuli and the individual's capacity to derive gratification from them (Agrawal et al. 2022; Eastwood et al. 2012; Wolff et al. 2024). Functionally, boredom serves as an internal signal that directs cognitive resources toward more relevant or motivating activities, indicating the need to reallocate attention to more stimulating or meaningful tasks (Bench and Lench 2013; Eastwood et al. 2012).

Boredom can be conceptualized as state boredom, arising in specific monotonous or non‐stimulating situations, and trait boredom, reflecting a chronic propensity to experience boredom across contexts (Bench and Lench 2013; Eastwood et al. 2012; Mercer‐Lynn et al. 2014; Seiler and Dan 2024; Tagliaferri et al. 2025). In any case, these constructs are positively correlated: individuals prone to boredom tend to experience situational boredom more intensely (Tagliaferri et al. 2025).

The conceptual claim advanced here is not that boredom is wholly independent of established psychiatric dimensions. Its proposed core feature is the aversive desire to be engaged together with a perceived inability to establish or sustain satisfactory engagement (Eastwood et al. 2012; Westgate and Wilson 2018). It is important to distinguish boredom from related psychopathological constructs. Boredom differs in emphasis from apathy, which is defined primarily by diminished motivation and initiation of goal‐directed behavior, and from anhedonia, which concerns reduced anticipatory or consummatory pleasure. Mind wandering denotes a shift toward task‐unrelated or self‐generated thought and may occur without aversive affect or a wish to re‐engage; fatigue is centered on perceived effort or depletion and commonly motivates rest; low mood is a broader affective state; and behavioral disengagement is an observable outcome that does not identify a specific subjective experience. Nevertheless, boredom covaries substantially with apathy, anhedonia, depression, attentional failure, and impulsivity, and the available evidence for discriminant validity is based largely on cross‐sectional self‐report and a limited number of psychometric or phenomenological studies (Y. K. Goldberg et al. 2011; Yeung et al. 2024). The transdiagnostic term is therefore used descriptively to indicate that boredom varies dimensionally and is observed across diagnostic categories.

According to the psychological perspective, boredom would emerge from the interaction between environment, attention, motivation, and perceived meaning. The Meaning‐and‐Attentional Components (MAC) model highlights that boredom results from an imbalance between the attentional demands of a task and its perceived subjective value: activities perceived as meaningless generate boredom, whereas a balance between attention and meaning can lead to a state of flow (Eastwood et al. 2012; Tagliaferri et al. 2025). The mismatch hypothesis also suggests that boredom occurs when a task is either too simple (understimulation) or too complex (overstimulation), creating a misalignment between attentional capacity and task demands (Berlyne 1960; Raffaelli et al. 2018). During boredom, task‐unrelated thoughts increase, and time is often perceived as passing slowly, which affects motivation and pleasure (Bench and Lench 2013; Eastwood et al. 2012; Raffaelli et al. 2018).

Boredom consistently presents negative affective valence, but physiological arousal can vary: it may manifest with low arousal, as in apathetic individuals, or with high arousal, as in agitated individuals who actively seek new stimuli (Critcher and Gilovich 2010; Eastwood et al. 2012; Goetz et al. 2014; Mikulas and Vodanovich 1993; Raffaelli et al. 2018). Greenson (1953) identified two main subtypes of boredom‐prone individuals: apathetic boredom‐prone, characterized by disinterest and attentional lapses, and agitated boredom‐prone, motivated to engage, albeit unable to find sufficiently rewarding stimuli, often associated with attention‐deficit/hyperactivity disorder (ADHD) symptoms (Greenson 1953; Malkovsky et al. 2012). These subtypes also differ in error sensitivity: apathetic individuals slow their responses after an error, indicating greater awareness of attentional lapses, whereas agitated individuals show lower awareness, similar to patients with traumatic brain injuries (Malkovsky et al. 2012; Molenberghs et al. 2009; Ndetei et al. 2023; Robertson et al. 1997).

Different data show that boredom engages a distributed brain network including the prefrontal cortex, parietal cortex, insula, basal ganglia, and cerebellum (Eastwood et al. 2012; Maggini 2000). Experimental paradigms inducing boredom also reveal involvement of the default mode network (DMN) and parietal and parieto‐occipital regions, which are implicated in attention regulation, executive processes, motivation, and emotional evaluation, suggesting that boredom is an active and dynamic phenomenon rather than a mere absence of stimulation (Chen and Rau 2022). EEG evidence indicates increased alpha activity and reduced beta activity during boredom, supporting the attentional failure hypothesis (Fakhr Tabatabaie et al. 2014; Raffaelli et al. 2018). Behaviorally, boredom drives the search for more intense or meaningful stimuli, which may include risky behaviors such as gambling, intense social experiences, or problematic use of the Internet and social media (Bench and Lench 2013; Tagliaferri et al. 2025).

From an adaptive perspective, boredom can be considered a functional signal that promotes exploration, learning, behavioral flexibility, and creativity (Elpidorou 2018; Seiler and Dan 2024; Wolff et al. 2024). Acting as a “warning signal,” it encourages individuals to explore new contexts, modify behavior, and seek more rewarding activities, facilitating learning and adaptation (Das et al. 2015; Mason and Brady 2009; Raz 2013; Sahoo et al. 2022; Seiler and Dan 2024).

In clinical contexts, state boredom is strongly associated with current psychopathological load and can vary depending on disorder and environmental context (Seiler et al. 2023). In closed psychiatric wards, boredom levels are higher compared to open wards, and the trajectory of boredom during hospitalization differs between diagnoses: psychotic patients tend to show increasing boredom, whereas patients with depression or addictions show decreasing levels. Trait boredom, instead, is associated with stable psychopathological symptoms such as depression, anxiety, impulsivity, sensation‐seeking, and addictive behaviors (Mercer‐Lynn et al. 2014; Raffaelli et al. 2018; Seiler et al. 2023; Tagliaferri et al. 2025). Among young people, boredom mediates the relationship between difficulties in emotion regulation and problematic behaviors, both internalizing and externalizing, with impulsivity increasing vulnerability to maladaptive social media use (Biolcati et al. 2018; Ndetei et al. 2023; Tagliaferri et al. 2025).

In summary, boredom is a complex and multifactorial phenomenon involving cognitive, affective, behavioral, neurobiological, and contextual aspects. Although often perceived as negative, it plays a fundamental adaptive and motivational role by stimulating exploration, creativity, and behavioral adaptation. Understanding boredom requires an integrated approach and can inform preventive strategies and therapeutic interventions, including CBT, mindfulness, empowerment, environmental enrichment, and personalized psychosocial therapies, aimed at reducing its negative impact and promoting well‐being and resilience (Bench and Lench 2013; Cannito et al. 2023; Chen and Rau 2022; Eastwood et al. 2012; Raffaelli et al. 2018).

This article aims to examine alterations in boredom across major psychiatric disorders, with a focus on their neurological and neurobiological correlates. It seeks to highlight the role of boredom in psychopathology and its potential as a biomarker and therapeutic target, and to explore how disruptions in the neural and cognitive mechanisms underlying boredom contribute to symptomatology and overall functioning.

## 2 Methods

This article was designed as a structured narrative review. Literature searches were conducted in PubMed/MEDLINE, Scopus, and Google Scholar and covered records indexed from database inception through March 2026. Search strategies were adapted to each database and combined terms related to boredom (“boredom,” “boredom proneness,” “state boredom,” and “trait boredom”) with terms related to psychiatry and psychopathology, including specific clinical conditions (e.g., ADHD, depression, anxiety, psychosis, schizophrenia, substance use and behavioral addictions, eating disorders, personality disorders, suicidality, and psychiatric hospitalization). Additional search terms covered neurophysiology, electroencephalography, neuroimaging, autonomic physiology, biomarkers, assessment, questionnaires, ecological momentary assessment, and experience‐sampling methods. The reference lists of relevant reviews and included articles were also examined to identify additional eligible studies.

Peer‐reviewed studies involving human participants were considered when boredom was explicitly assessed as a state or trait, or experimentally induced, and examined in relation to psychiatric symptoms or diagnoses, neurophysiological or neuroimaging outcomes, autonomic measures, functioning, treatment‐related variables, or clinically relevant behaviors. Conceptual articles and reviews were included when they contributed to the definition, theoretical understanding, or measurement of boredom. Studies were excluded when boredom was not directly assessed or induced, the term was used only colloquially, or the reported outcomes fell outside the scope of the review.

Given the heterogeneity of the populations, measures, experimental paradigms, and outcomes, the evidence was synthesized narratively rather than pooled quantitatively. Divergent findings were evaluated by considering study design, sample characteristics, state‐versus‐trait operationalization, induction paradigm, comparator condition, covariate adjustment, and the extent to which conclusions were supported by longitudinal, experimental, or intervention evidence. Greater interpretive weight was assigned to designs capable of addressing temporal or causal relationships than to cross‐sectional associations. As this was not a systematic review, no formal risk‐of‐bias instrument was applied; consequently, incomplete retrieval and selection bias cannot be excluded.

### 2.1 Neurophysiology of Boredom

#### 2.1.1 Oscillatory Dynamics and Connectivity During Boredom (EEG Studies)

Studying boredom poses methodological challenges, as it is predominantly an internal and subjective experience, making it difficult to determine its precise onset and offset. Although the application of time‐sensitive neurophysiological methods such as EEG is complex, a growing body of research has identified reliable associations between neural oscillatory activity and subjective experiences of boredom (Raffaelli et al. 2018).

Early EEG investigations were among the first to propose neurophysiological correlates of boredom, suggesting that increases in alpha activity during mentally unstimulating tasks reflect both visual inattention and the subjective experience of boredom (Oswald 1962). Subsequent studies confirmed that, although alpha power can index different arousal levels and functional brain states, boredom is consistently associated with enhanced alpha activity, occurring under both low and high arousal conditions (Fahlman et al. 2013). More fine‐grained frequency analyses revealed additional oscillatory signatures of boredom. Beta‐2 power in the dorsolateral prefrontal cortex (DLPFC) decreases during exposure to self‐chosen boring music compared with engaging music, indicating lower cortical activation in response to uninteresting stimuli (Tabatabaie et al. 2014). Moreover, theta power over midline electrodes has been reported to correlate with boredom, with increases in theta synchronization observed just prior to participants’ subjective reports of boredom (Miyauchi and Kawasaki 2018). Posterior alpha power is also shown to be increased during peak boredom, and converging evidence suggests that enhanced midline alpha and frontal theta activity may reflect DMN‐related engagement during periods of maximal boredom and mind‐wandering (Esposito et al. 2022; Miyauchi and Kawasaki 2018).

Frontal EEG markers further highlighted the involvement of motivational and regulatory processes during boredom. In particular, frontal alpha asymmetry (FAA) has been shown to vary as a function of boredom proneness: individuals reporting higher levels of boredom in daily life exhibited a rightward shift in FAA during monotonous tasks (Perone et al. 2019). Greater intra‐individual variability in FAA and in the frontal theta/beta ratio was also associated with higher boredom and reduced enjoyment, suggesting dynamic fluctuations in attentional and motivational control (Perone et al. 2021). Age‐dependent effects have also been reported, with younger adults showing a relative reduction in left frontal engagement during boredom, whereas older adults displayed a less lateralized pattern, indicating that frontal cortical involvement varies across the lifespan (Nettinga et al. 2024).

Experimentally induced boredom has been further linked to reduced cortical engagement and diminished cognitive control, as reflected by increased alpha power and decreased frontal theta activity, and attenuated event‐related potentials, including reduced P3 amplitudes and error‐related responses during task performance (Yakobi et al. 2021). Consistent with these findings, studies using prospective memory paradigms have reported increased alpha power in parietal and parieto‐occipital regions during boredom, with bottom‐up boredom conditions eliciting higher alpha power than top‐down approaches, reflecting differences in the processing of boredom (Chen and Rau 2022, 2023).

Beyond local oscillatory changes, recent EEG evidence indicates that boredom is also associated with altered functional connectivity and large‐scale network organization (Yuvaraj et al. 2025). Experimentally induced boredom was linked to increased global efficiency, elevated clustering, and higher local efficiency across alpha, beta, and gamma bands, alongside decreased characteristic path length, suggesting a distinct and integrated pattern of brain network activity during the bored state (Yuvaraj et al. 2025).

#### 2.1.2 Neural Network Correlates of Boredom (fMRI Studies)

Early evidence on the neural mechanisms underlying boredom highlights the involvement of frontal and prefrontal regions that are central to attentional processes (Eastwood et al. 2012). Converging findings indicate that boredom is closely linked to the DMN, which includes core hubs such as the posterior cingulate cortex (PCC), precuneus, and ventromedial prefrontal cortex (vmPFC), as well as lateral cortical regions including the inferior parietal lobule (IPL) and temporoparietal junction (TPJ) (Buckner et al. 2008; Danckert and Merrifield 2018). The DMN supports introspective, mnemonic, and internally oriented cognitive processes and shows heightened activity during boredom, when attention shifts from external stimuli toward internal mental processes (Christoff et al. 2016; Danckert and Merrifield 2018). However, this pattern should not be interpreted as a neural signature specific to boredom. The DMN supports several forms of internally oriented cognition, including mind wandering, autobiographical memory, self‐generated thought, and reduced attention to external stimuli (Buckner et al. 2008; Christoff et al. 2016). Increased DMN engagement during boredom may therefore reflect a broader shift from externally directed attention toward internal mentation or reduced task engagement, rather than boredom itself. Its interpretation depends on the induction paradigm, task demands, and comparator condition.

Structural neuroimaging evidence suggests that individual differences in susceptibility to boredom may be partly explained by variability in DMN‐related regions. Voxel‐based morphometry (VBM) analyses revealed a negative correlation between boredom proneness and gray matter volume in the precuneus and cuneus, suggesting that reduced structural integrity in these areas may underlie predisposition to boredom (Wang et al. 2021). Experimental induction of boredom through sustained attention tasks has been associated with increased DMN activity, particularly in the precuneus, PCC, and mPFC, alongside decreased engagement of fronto‐parietal control and salience networks, including the anterior insula and anterior cingulate cortex (ACC) (Danckert and Merrifield 2018). This pattern reflects a dominance of internally oriented cognition and reduced externally directed attentional control (Danckert and Merrifield 2018). Importantly, analyses of network dynamics indicate that boredom is accompanied by greater variability and reduced stability of DMN connectivity over time, highlighting dynamic fluctuations that may correspond to an internal search for cognitive stimulation (Danckert and Merrifield 2018). Overall, DMN involvement appears to be a recurrent but non‐specific correlate of boredom‐induction paradigms.

Functional neuroimaging evidence also indicates that boredom would emerge from distinct neural configurations, despite comparable subjective experiences (Danckert and Merrifield 2018). Resting state, sustained attention tasks, and boredom‐inducing video viewing elicited similar levels of self‐reported boredom, yet were characterized by different patterns of functional coupling between the anterior insula and the DMN (Danckert and Merrifield 2018).

Additional task‐based evidence shows that boredom, operationalized as a reduction in affective valence combined with prolonged goal‐undirected activity, was associated with bilateral activation of the vmPFC and insula, alongside deactivation of the right precuneus and hippocampus during video game play (Mathiak et al. 2013).

Similarly, boredom relative to flow during sustained attention tasks was characterized by increased activation of DMN hubs (PCC/precuneus and mPFC) and reduced engagement of fronto‐parietal attention networks, accompanied by performance decrements indicative of attentional disengagement (Ulrich et al. 2014, 2016). Boredom relative to flow was also linked to increased activation in medial temporal lobe (MTL) regions, including the left amygdala, hippocampus, and parahippocampal gyrus, together with the mPFC (Ulrich et al. 2014). Resting‐state functional connectivity (RSFC) analyses further support the role of DMN hubs in boredom proneness (Wang et al. 2021). Functional connectivity between the precuneus/cuneus and PCC was positively associated with boredom proneness (Wang et al. 2021). Mediation analyses indicated that boredom proneness fully mediated the relationship between this connectivity pattern and procrastination, highlighting the precuneus as a critical neural substrate underlying individual differences in boredom susceptibility (Wang et al. 2021). Overall, converging neuroimaging evidence indicates that increased engagement of the DMN represents the most consistent neural correlate of boredom, particularly involving the mPFC, PCC/precuneus, and MTL (Raffaelli et al. 2018). Activity in other regions, such as the hippocampus and anterior insula, emerges as a significant but less consistent correlate across studies (Danckert and Merrifield 2018; Mathiak et al. 2013; Raffaelli et al. 2018; Ulrich et al. 2014, Ulrich et al. 2016).

The involvement of the vmPFC during boredom may partly reflect its role in evaluating internally generated thoughts and self‐related content (Raffaelli et al. 2018). Distinct PFC subregions support specific evaluative processes, with the medial orbitofrontal cortex implicated in the evaluation of internal events and the rostromedial PFC in self‐related appraisal (Dixon et al. 2017). This pattern is consistent with the internally focused, self‐referential cognition typically elicited when individuals are unable to engage with external tasks (Raffaelli et al. 2018). Alternatively, it has been interpreted as reflecting a shift of attention toward internally oriented cognition, potentially serving as a compensatory response to disengagement from external tasks, consistent with the involvement of the MTL and vmPFC in spontaneously generated thought (Ellamil et al. 2016).

#### 2.1.3 Autonomic Markers of Boredom

Data on autonomic arousal indicate that boredom is not uniformly characterized by a low‐arousal state, but is associated with heightened autonomic activation (Raffaelli et al. 2018). In particular, it has been related to increased markers of autonomic arousal, including heart rate (HR) and electrodermal activity (EDA) (Raffaelli et al. 2018). Consistent with this view, experimental studies report increased EDA during boredom induced by sustained attention tasks, as well as elevated HR during monotonous and highly repetitive activities, such as repeated writing tasks (London et al. 1972; Lundberg et al. 1993; Ohsuga et al. 2001).

At the same time, some studies suggest that boredom may modulate autonomic responses in divergent ways, depending on the physiological index considered (Raffaelli et al. 2018). A decrease in EDA has been reported during boredom (Frith and Allen 1983; O'Connell et al. 2008), while HR has increased (Coles 1972). Similarly, monotonous and minimally stimulating states commonly interpreted as boredom were associated with increased respiratory sinus arrhythmia (RSA) and longer cardiac periods, reflecting reduced arousal and decreased attentional engagement (Pattyn et al. 2008).

More recent work further emphasized the dynamic nature of autonomic responses during boredom. Experimentally induced boredom was associated with reduced tonic autonomic arousal, as shown by lower HR and EDA, alongside increased intra‐individual physiological variability over time (Clay et al. 2024). This pattern suggests oscillations between disengagement and transient micro‐activations, rather than sustained hypo‐ or hyperarousal (Clay et al. 2024). In line with this interpretation, boredom‐related effort during monotonous cognitive tasks was positively associated with elevated EDA, indicating that sympathetic activation may accompany attempts to maintain engagement (Radtke et al. 2025).

Taken together, these findings indicate that boredom cannot be characterized by a single autonomic profile. While several studies point to elevated arousal and sympathetic activation, other data report markers of reduced arousal to suggest that boredom may encompass both low‐ and high‐arousal states (Eastwood et al. 2012; Martin et al. 2006; Mikulas and Vodanovich 1993; Vodanovich 2003). Conflicting results likely reflect the difficulty of distinguishing boredom from low‐arousal apathy; nonetheless, the most robust evidence points to boredom being characterized primarily by high arousal and sympathetic activation (Bench and Lench 2013).

Beyond cardiovascular and electrodermal measures, boredom is reliably associated with ocular markers of attentional disengagement. Increased blink rates reliably distinguish bored from attentive states, with individuals high in boredom proneness exhibiting elevated blink frequency (Danckert et al. 2018; Esposito et al. 2022). Moreover, variations in subjective boredom are best predicted by a combination of blink rate and frontal theta synchronization, highlighting the convergence of autonomic and cortical indices (Danckert et al. 2018; Esposito et al. 2022).

Overall, autonomic evidence supports the view that boredom is not a stationary physiological state, but rather one characterized by dynamic fluctuations and instability across autonomic and cortical processes over time (Clay et al. 2024; Danckert et al. 2018; Yakobi et al. 2021).

### 2.2 Boredom in Psychiatric Disorders

In recent years, the construct of boredom has been reevaluated within the scientific literature, elevating it from a transitory and marginal state to a central clinical factor that may negatively affect mental health, global functioning, and therapeutic outcomes (Y. K. Goldberg et al. 2011). From the phenomenological point of view, this experience is clearly distinct from apathy and anhedonia (Yeung et al. 2024) that takes the form of a motivational paradox in which the persistent aspiration for cognitive engagement clashes with the functional inability to achieve it (Constant et al. 2021; Eastwood et al. 2012). This dyscrasia generates a complex aversive experience including a deep sense of emptiness, environmental disconnection, and a perception of the irrelevance of available stimuli (Steele et al. 2013). According to the neurocognitive perspective, boredom is intrinsically associated with deficits in emotional regulation, sustained attention, and motivational processes (Isacescu et al. 2017). Contrary to the common association with states of low arousal, pathological boredom often appears as restlessness and agitation, which signal a systemic inability to modulate attention toward internal or external stimuli. It is, therefore, crucial to recognize the predisposition to boredom, a stable and context‐independent propensity that acts as a powerful factor in the maintenance of psychopathology and a precursor to maladaptive behaviors adopted to mitigate the associated distress (Iannattone et al. 2024). As such, boredom may be considered a transdiagnostic feature that permeates heterogeneous clinical conditions, while also representing a factor in worsening prognosis (Binnema 2004). Therefore, an analytical understanding of these manifestations and their underlying mechanisms appears essential for designing targeted therapeutic approaches in different psychopathological conditions.

#### 2.2.1 Attention Deficit/Hyperactivity Disorder

In ADHD, boredom is frequently reported as a correlate of executive dysfunction and the neurocognitive dynamics of the disorder (Orban et al. 2026). It results from a complex interaction between deficits in attentional regulation, intolerance of anticipation, and a need for immediate stimulation resulting from a reduced ability to extract information from the environment (Seiler et al. 2025). This is subjectively perceived as intense boredom that triggers impulsive sensation‐seeking reactions aimed at compensating for the hypostimulation (Hunter and Eastwood 2018). Executive functions mediate this relationship, in particular attentional control, working memory and delay aversion, a neurobiological difficulty tolerating waiting (Hsu et al. 2025; Van Dessel et al. 2018). Phenomenologically, the apathetic phenotype, associated with pure disengagement and attention deficits, or the agitated variant, characterized by motor restlessness and reduced sensitivity, are typical signals of boredom (Wilbertz et al. 2012). This variant reflects a motivation to act, frustrated by deficits in reward evaluation (Spaeth et al. 2015), and it shows the most significant correlations with hyperactivity and impulsivity in adulthood (Y. Goldberg and Danckert 2013; Kass et al. 2003; Malkovsky et al. 2012). From a therapeutic perspective, the only pharmacotherapy that has specifically been shown to be effective in leading to a reduction in perceived boredom, through the improvement of sustained attention, is methylphenidate, although discontinuation of treatment leads to a rapid restoration of baseline levels of boredom and the consequent risk of sensation‐seeking behaviors, highlighting the importance of ongoing clinical management (Golubchik et al. 2020, Golubchik et al. 2021). Another potentially promising drug, but one that does not yet have specific evidence regarding boredom, is lisdexamfetamine dimesylate, which, however, has been shown to induce a reduction of up to 30% of the symptoms typical of the inattentive phenotype in adult patients with ADHD (Newcorn et al. 2017; Soutullo et al. 2013). On the other hand, the efficacy of atomoxetine is expressed mainly on the neural basis of agitated boredom and aversion to delay, strengthening the inhibitory executive architecture, thus allowing the patient to rationally modulate and contain the activation triggered by delays and waiting, no longer seeking immediate and impulsive gratification to repress boredom, but becoming capable of tolerating the emotional distress of delay (Bush 2010). From this perspective, although the pharmacological component is crucial, it alone cannot permanently rewrite dysfunctional neural architectures nor provide patients with metacognitive strategies to tolerate low‐informational entropy environments, making it extremely important to integrate it with cognitive therapy protocols, which present convincing evidence of short‐ and long‐term efficacy in the treatment of ADHD and consequently in the management of associated boredom (X. Yang et al. 2025).

#### 2.2.2 Mood and Anxiety Spectrum Disorders

The relationship between boredom and affective disorders represents a highly complex clinical domain, in which this construct is not limited to the role of an ancillary symptom, but emerges as a potential statistical mediator in observational studies of psychological distress and impaired quality of life (Hofer et al. 2022; Schwartze et al. 2021). Evidence highlights a significantly stronger link between the experience of boredom and depressive symptoms than that observed with other negative affective states (Eckland et al. 2022), identifying boredom proneness as the primary mediating factor between the perception of stress and the resulting emotional maladjustment (Lee and Zelman 2019; Yan et al. 2021). The parallel improvement in mood and perceived boredom in response to serotonin reuptake inhibitors (SSRIs) supports the coherence of this conceptualization (Theobald et al. 2003). Boredom plays a central role in depressive symptoms, in which states of understimulation may lead individuals to impulsively seek overstimulation to fill feelings of emptiness and immobility (Chiappini et al. 2025).

This vulnerability extends to anxiety disorders (Silove et al. 1997), in which anxious avoidance generates monotony that fuels boredom, which in turn exacerbates rumination and internal tension (Danckert and Merrifield 2018; Zhao et al. 2024). Therefore, routine aversion emerges as a shared phenotypic trait between anxiety and depression (Boschloo et al. 2013). The clinical impact of this construct is also evident in responses to environmental failures, such as job boredom, which correlates with depression and anxiety when not counteracted by proactive job crafting strategies (J. Li et al. 2024; Phomprasith et al. 2022; van Hooft and van Hooff 2018). During the COVID‐19 pandemic, boredom has been identified as a major mediator of increased depression and anxiety (Weiss et al. 2022; M. Yang et al. 2022) through a subjective perception of time slowing down and the inability to channel resources toward active functioning.

#### 2.2.3 Psychotic Spectrum and Related Disorders

In psychotic spectrum disorders, boredom is not a mere absence of activity, but rather an active and insidious clinical condition, defined as a state of generalized hypohedonia (Todman 2003). Although historically associated with negative symptoms, the subjective experience of boredom is partially distinct from apathy and avolition due to the presence of a component of anxiety, restlessness, and active dissatisfaction (Ricci et al. 2025). The impact of this experience on global functioning appears pervasive, as proneness boredom in schizophrenic patients is significantly higher than in the general population, directly correlating with a marked reduction in quality of life and perceived well‐being (Gerritsen et al. 2015). Recent evidence suggests that trait boredom correlates positively with dimensions of negative schizotypy and a reduced sense of purpose, acting as a marker of weakness that may precede the onset of overt pathology (McGough et al. 2025). A clinical issue of primary importance concerns the interaction between boredom and pharmacological compliance, as the stability induced by antipsychotic treatment is often experienced as an intolerable dulling of life, leading to abandonment of treatment to paradoxically seek the stimulation associated with the acute phase (Branković 2015). It is therefore essential that treatment systematically integrate occupational rehabilitation interventions, social skills training, and coping strategies aimed at structuring time and managing pleasure, to preserve the therapeutic alliance and prevent relapse.

#### 2.2.4 Substance Abuse and Behavioral Addictions

As we've seen previously, boredom has been increasingly recognized in recent years as a psychological construct with important implications for mental health and maladaptive behaviors (Tam, van Tilburg, and Chan 2021). It has been found to be associated with dysregulation of reward processing and motivational systems, particularly those involving dopaminergic pathways related to novelty seeking and reinforcement learning (Costa et al. 2014). This often leads individuals to seek out novel, stimulating, or risky experiences aimed at resolving the mismatch between cognitive resources and environmental stimuli, prompting them to seek alternative activities capable of restoring optimal levels of arousal and engagement (Bench and Lench 2019; Matijašević et al. 2025). In these individuals, boredom therefore leads to the development of maladaptive coping strategies, such as compensatory mechanisms to counteract understimulation and internal distress (Mercer‐Lynn et al. 2014). These include sensation‐seeking behaviors and impulsive, reward‐oriented decisions. These are widely recognized risk factors for the development of addictive disorders, including substance use and behavioral addictions, which can serve as a rapid means of alleviating aversive internal states (Iso‐Ahola and Crowley 1991).

Analysis of pathological addictions reveals a critical intersection between boredom proneness and vulnerability to substance abuse and behavioral addictions, outlining a framework in which impulsivity and sensation seeking serve as coping strategies against aversive internal states (X.‐J. Yang et al. 2020). Evidence shows that alcohol and drug use appears to be driven by a need to alleviate the symptoms of negative affect (Del Boca and Hesselbrock 1996; Kolas and von Mühlenen 2025; Mintz et al. 1979; Oh et al. 2019). Similarly, tobacco data indicate that smokers seek it to reduce negative effects and increase stimulation (Fatani et al. 2022; Weinberger et al. 2011). During lockdown periods, the pandemic has accelerated these dynamics, providing empirical evidence on the impact of social isolation and enforced boredom on the significant increase in the consumption of alcohol, cannabis, and even legal stimulants such as caffeine and theine, driven by the need to manage negative affectivity and excessive unstructured time (Castellana et al. 2021; Liang et al. 2022; Mehra et al. 2023; Merino‐Casquero et al. 2025; Stack et al. 2021; Weerakoon et al. 2021).

Trait boredom has been recognized as a key factor in the emergence of new digital addictions, acting as a mediating variable in problematic internet (PIU), social media, and smartphone use (Tagliaferri et al. 2025). Especially among young people, the digital world acts as a compensatory factor for lack of stimulation, triggering a sequential pathway in which boredom proneness predicts technology addiction, which, in turn, exacerbates sleep procrastination and emotional instability (W. Li et al. 2015; Marazziti et al. 2014). The interaction between sensation seeking and boredom peaks in adolescence, associated with externalizing behaviors and compulsive viewing of short‐form video formats, although the phenomenon is also found in the adult population (Baltacı and Açar 2025; Freund et al. 2021; Jahagirdar et al. 2024). Loneliness and dysfunctional family contexts act as further promoting factors for these outcomes (Orsolini et al. 2023; Pop‐Jordanova 2024; Ventriglio et al. 2024). This dynamic, which affects different age groups, sees online gambling and slot machine use as a powerful factor promoting addiction thanks to visual stimulation and immediate gratification, which emerge as predictors of problematic behaviors related to boredom susceptibility and disinhibition (Biolcati et al. 2018; Bonnaire and Barrault 2018; Kruger et al. 2022; McNeilly and Burke 2000). Indeed, gambling and compulsive media use have also been fueled by susceptibility to boredom and intolerance of uncertainty, with ambivalent effects in the older population, where technology has partially mitigated boredom without, however, resolving the underlying loneliness (Chao et al. 2020; Dura‐Perez et al. 2022; Fluharty et al. 2022; Marchetti et al. 2023).

Overall, these findings highlight boredom as a multifaceted psychological construct, a candidate vulnerability factor or proximal trigger for both the development and maintenance of addictions (van Tilburg and Igou 2012). It plays a key role in motivational regulation and maladaptive coping processes, placing it at the intersection of emotional dysregulation, reward‐seeking behavior, and vulnerability to addictive patterns (Z. Yang et al. 2026). Within this conceptual framework, investigating the relationship between boredom proneness and addictive behaviors has become increasingly relevant for understanding the pathogenesis of pathological addictions in different populations and sociocultural contexts (Biolcati et al. 2018; X.‐J. Yang et al. 2020). All of this elevates boredom to a priority therapeutic target, promoting interventions aimed at strengthening attention regulation, tolerance for monotony, and engagement in activities with subjective meaning to discourage addictions and prevent relapse.

#### 2.2.5 Eating Disorders

Within eating behaviors, boredom appears to be proximal antecedent capable of triggering emotional eating (Armitage 2015; Schulte 2016). This dynamic extends to severe psychopathological conditions such as binge eating disorder (BED), in which boredom represents one of the most intense antecedents of binge eating episodes, with an impact comparable to body dissatisfaction (Stickney and Miltenberger 1999). Similarly, in complex medical populations such as patients undergoing bariatric surgery, boredom interacts with food cravings and availability, triggering binge eating episodes, followed by feelings of guilt or disgust (Yu et al. 2023). Boredom‐induced eating underlies specific difficulties in emotional regulation, characterized by a reduced understanding of one's internal states and deficits in goal‐directed behaviors (Braden et al. 2018). Boredom thus operates through a mechanism of unawareness and a hedonic search aimed at filling the experiential void (Braden et al. 2025; Yu et al. 2023). Therapeutic implications highlight the need for differentiated interventions for non‐clinical samples for which cognitive strategies are effective (Armitage 2015), while in clinical and post‐bariatric settings, a personalized approach that integrates boredom as a potentially therapeutic target is essential to prevent weight regain (Braden et al. 2023; Yu et al. 2023).

#### 2.2.6 Borderline Personality Disorder

Boredom emerges as a persistent clinical experience that goes beyond mere inactivity in personality disorders characterized by affective instability, particularly in borderline personality disorder (BPD). Although excluded from the formal diagnostic criteria of the DSM‐IV due to the lack of unequivocal empirical data, contemporary theoretical models highlight how reactivity to boredom interacts with identity instability and chronic feelings of emptiness, triggering impulsive or self‐destructive behaviors aimed at regulating an otherwise intolerable internal state (Masland et al. 2020). However, the literature has imposed a rigorous differentiation between emptiness, which, along with fear of abandonment and despair, shows an elective correlation with BPD (Fulham and Fitzpatrick 2025; Miller et al. 2020), and boredom that represents a qualitatively distinct construct and is less associated with these feelings (Klonsky^2008^). Despite this taxonomic separation in adults, boredom regains a central psychopathological role in adolescence, where it acts as a precursor to internal tension and acting out (Freund et al. 2021; Schwartze et al. 2020). During this developmental stage, the experience of boredom and anhedonia, often associated with dysthymic states, serves as a catalyst for antisocial behaviors, substance abuse, and self‐harm, used as strategies to interrupt aversive stasis (Iannattone et al. 2024; James et al. 1996). Ultimately, boredom in BPD may be defined as an active driver of behavioral dysregulation and pathological stimulation seeking, while maintaining its clinical autonomy from existential emptiness (Miller et al. 2021).

#### 2.2.7 Suicidal Ideation and Behaviors

Boredom may represent a crucial and independent risk factor for suicidal ideation (Lissak et al. 2024). This nosological autonomy has its historical roots in the concept of tedium, understood as physical and emotional exhaustion, which correlates significantly with current suicidal preoccupation, especially in subjects with a history of previous attempts at suicide (Nederkoorn et al. 2016). Studies conducted on patients with major depression revealed that boredom, frequently associated with feelings of tension and sadness, is the only significant predictor of the onset of suicidal thoughts in the hours immediately following suicide, even surpassing established constructs such as hopelessness in prognostic efficacy (Ben‐Zeev et al. 2012). This causal relevance is corroborated by the subjective perception of at‐risk youth populations who tend to attribute the suicidal act to an existential emptiness and a lack of stimulating activities rather than to mental illness itself (Heled and Read 2005). In contexts of forced isolation, boredom can trigger dysfunctional coping mechanisms such as maladaptive daydreaming, which is used as a form of self‐medication to regulate stress but worsens psychosocial distress (Somer et al. 2020).

#### 2.2.8 Boredom in Inpatient Psychiatric Units

Psychiatric hospitalization represents a particularly critical clinical context, in which the interaction between individual psychopathological vulnerability and the structural rigidity of the institution fosters a marked exacerbation of boredom (Tamarelli et al. 2024). This experience, characterized by a profound deprivation of meaning and emotional impoverishment resulting from the patient's inability to engage in subjectively valuable activities, appears to exhibit no significant differences across diagnostic categories, thus constituting a transversal and ubiquitous phenomenon, independent the admission diagnosis (Newell et al. 2012; Seiler et al. 2025). This phenomenological homogeneity suggests that hospital boredom is primarily due to intrinsic characteristics of the care environment, such as sensory hypostimulation, monotony, and a drastic reduction in personal agency (Binnema 2004; Björkdahl et al. 2016). These findings require moving beyond operational paradigms based on filling‐time interventions, orienting services toward interventions that promote authentic participation and restore significant decision‐making power to the patient (Steele et al. 2013).

#### 2.2.9 Clinical and Therapeutic Implications

The clinical implications of boredom are not limited to mere subjective distress, significantly impacting the effectiveness of interventions and long‐term patient management (Seiler et al. 2023). In the therapeutic setting, this construct emerges as a critical predictor of dropout and reduced medication adherence (Gerritsen et al. 2015). At the same time, institutional boredom in inpatient settings represents a clinical variable that fuels chronicity and erodes the cognitive and motivational reserves necessary for the therapeutic alliance (Binnema 2004; Steele et al. 2013). This state of hypostimulation not only aggravates psychopathology and healthcare costs but can also degenerate into impulsive behaviors, escape attempts, or escalating aggression (Newell et al. 2012; Seiler et al. 2023). Diagnostically, it is necessary to recognize the phenomenological autonomy of boredom within nosological classification systems, integrating routine assessment with psychometric tools to distinguish this state from apathy and anhedonia (Gorelik and Eastwood 2024). Boredom requires specific rehabilitation strategies aimed at rebuilding the capacity for active engagement and tolerance of inactivity (Seiler et al. 2023). Identifying this construct would allow to explain several cases of apparent drug resistance (Branković 2015). Therefore, boredom requires an integrated approach, capable of combining the restoration of attentional circuits with the reconstruction of subjective meaning, elevating it to a central target of precision medicine in psychiatry (Ndetei et al. 2023).

Treating pathological boredom requires adopting a multimodal intervention paradigm that integrates psychotherapeutic strategies, targeted pharmacological approaches, and environmental enrichment interventions (Steele et al. 2013). Within the psychotherapeutic framework, Cognitive Behavioral Therapy (CBT) aims to restructure negative appraisals related to inactivity and restore agency through behavioral activation (Dimidjian et al. 2011), while Acceptance and Commitment Therapy (ACT) promotes proactive engagement in value‐oriented activities to restore purpose and meaning to subjective experience (Hayes et al. 2006). In clinical settings characterized by marked emotional dysregulation or rumination, integrating protocols based on Dialectical Behavior Therapy (DBT) and Metacognitive Therapy (MCT) is crucial for providing mindfulness tools and techniques for distancing from dysfunctional thought patterns, thus preventing impulsive responses, self‐harm, or depressive tendencies (Kothgassner et al. 2021; Normann and Morina 2018). These coping skills also take on an essential protective value in the context of pathological addictions, reducing the automatic use of surrogate gratifications through greater tolerance of monotony and better attentional modulation (Wardle et al. 2024). From a pharmacological perspective, although there are no molecules with specific indications, optimizing dopaminergic transmission is the strategy of choice for addressing the neurobiological substrate of the disorder (Peng et al. 2024; Webber et al. 2021). At the same time, clinical management in inpatient settings must move beyond the logic of passive custody and toward structured environmental enrichment and occupational therapy interventions, prioritizing activities with subjective salience and promoting autonomy to mitigate iatrogenic boredom (Seiler et al. 2023). Ultimately, a personalized therapeutic approach requires constant monitoring of boredom levels as a clinical outcome parameter, ensuring a rehabilitation process that promotes functional recovery and the restoration of the vital bond between the individual and their environment (Ndetei et al. 2023).

## 3 Discussion

The present review highlights how boredom should no longer be considered a marginal or secondary phenomenon in psychopathology, but rather a complex experiential state emerging from the interaction between attentional regulation, motivational processes, and the perceived meaning of ongoing activities (Eastwood et al. 2012; Westgate and Wilson 2018). The evidence reviewed herein suggests that boredom reflects a condition in which individuals experience a persistent desire for cognitive engagement, although encountering difficulties in sustaining it. This results in an aversive state characterized by dissatisfaction, attentional disengagement, and a search for alternative stimulation (Ng et al. 2024).

It is worth noting that boredom appears to be conceptually and clinically distinct from apathy, as it involves a paradoxical condition in which motivational tension persists but cannot be effectively directed toward rewarding activities. On the contrary, apathy is characterized by reduced motivation and diminished goal‐directed behavior (Y. K. Goldberg et al. 2011; Yeung et al. 2024). This distinction is particularly relevant in psychiatric settings, as the behavioral consequences of boredom are often associated with active attempts to escape the aversive experience rather than passive disengagement.

From a neurobiological perspective, converging findings deriving from EEG, neuroimaging, and autonomic studies suggest that boredom is associated with alterations in large‐scale brain networks involved in attention, internally oriented cognition, and motivational processes (Danckert and Merrifield 2018; Raffaelli et al. 2018). In particular, increased engagement of the DMN together with reduced activation of fronto‐parietal control networks may reflect a shift towards internally focused cognition and mind‐wandering when externally directed attention cannot be maintained (Ulrich et al. 2014; Wang et al. 2021). Although DMN involvement is recurrent across several studies, it is shared with multiple forms of internally oriented cognition and reduced external engagement. Consequently, reverse inference from DMN activity to the subjective experience of boredom should be avoided. At the same time, oscillatory EEG findings, such as increased alpha activity and changes in theta‐related processes, are broadly consistent with the attentional failure hypothesis, while suggesting reduced task engagement and difficulties in maintaining cognitive control (Fahlman et al. 2013; Raffaelli et al. 2018).

Autonomic findings further indicate that boredom cannot be described as a simple low‐arousal condition. Rather, the literature suggests that it is a dynamic physiological state with fluctuations between disengagement and attempts to re‐engage with the task environment (Clay et al. 2024; Radtke et al. 2025). This variability may help explain the heterogeneous phenomenological presentations of boredom observed in clinical populations, ranging from apathetic disengagement to restless agitation (Mikulas and Vodanovich 1993).

From a clinical perspective, one of the most relevant findings emerging from the literature is the transdiagnostic nature of boredom. Evidence indicates that boredom proneness is associated with a wide range of psychiatric conditions, including ADHD, mood disorders, addictions, eating disorders, and personality disorders (Mercer‐Lynn et al. 2014; Ndetei et al. 2023; Seiler et al. 2025). Across these conditions, boredom appears to function as both a vulnerability factor and a mechanism contributing to the maintenance of psychopathology. In particular, the inability to tolerate prolonged states of low stimulation may promote compensatory behaviors aimed at increasing arousal or emotional intensity, such as impulsivity, sensation seeking, or engagement in addictive behaviors (Bench and Lench 2013; Tagliaferri et al. 2025). This interpretation is consistent with theoretical models conceptualizing boredom as a signal indicating that current activities fail to meet the individual's attentional or motivational requirements (Eastwood et al. 2012; Westgate and Wilson 2018). When adaptive regulation mechanisms function properly, boredom may encourage exploration and behavioral change. However, when regulatory processes are compromised, as they may occur in several psychiatric conditions, the same signal may contribute to maladaptive coping strategies and worsening clinical outcomes (Iannattone et al. 2024; Tam, van Tilburg, Chan, Igou, et al. 2021).

The relevance of boredom becomes particularly evident in institutional and inpatient psychiatric settings. Several studies reported elevated levels of boredom during hospitalization, largely due to environmental monotony, reduced autonomy, and limited opportunities for meaningful engagement (Binnema 2004; Björkdahl et al. 2016; Seiler et al. 2025). Under these circumstances, boredom may negatively affect treatment adherence, increase agitation or impulsive behaviors, and undermine therapeutic engagement (Gerritsen et al. 2015; Newell et al. 2012). These findings suggest that clinical management should not only focus on symptom reduction but also on promoting structured activities and environments capable of restoring meaningful engagement and a sense of agency in patients.

Overall, the available evidence supports the view that boredom may represent a relevant psychological and neurobiological construct with important implications for understanding and managing psychiatric disorders. Recognizing boredom as a clinically significant experience may contribute to a more comprehensive understanding of patient behavior and to the development of interventions aimed at improving engagement, emotional regulation, and adaptive functioning (Ndetei et al. 2023).

In any case, methodological challenges in the current scientific research on boredom in psychiatry, limit its generalizability to clinical practice (Vodanovich 2003; Vodanovich and Watt 2016). State boredom should be distinguished from trait boredom or boredom proneness. Although these dimensions are correlated, they are not interchangeable. Moreover, commonly used instruments, including the Multidimensional State Boredom Scale, the Boredom Proneness Scale, and its short form, differ in time frame, factor structure, and item content, while domain‐specific questionnaires and single‐item ratings introduce further heterogeneity (Fahlman et al. 2013). Laboratory inductions, including sustained‐attention tasks, repetitive activities, low‐stimulation videos, and waiting periods, also differ in cognitive load, duration, controllability, meaning, and arousal. These paradigms may elicit fatigue, frustration, low mood, mind wandering, or generic disengagement alongside boredom, thereby limiting construct specificity. Ecological momentary assessment and experience‐sampling methods provide greater temporal and contextual resolution but often rely on brief measures and remain affected by participant burden, reactivity, and differences in sampling schedules. Given the paucity of research, longitudinal and experimental studies are also needed to determine whether boredom acts as a primary casual factor in the psychopathological cascade, or whether it should be interpreted solely as an epiphenomenon (Danckert et al. 2018). A crucial aspect for the evolution of the field lies in moving beyond reliance on subjective self‐report instruments in favor of identifying objective biomarkers, such as electroencephalographic or neurophysiological parameters (Westgate 2020). From a neurobiological perspective, while evidence on DMN dysregulation is promising, further investigation through functional neuroimaging is needed to map the dynamics of the dopaminergic and serotonergic systems in relation to the cortisol response. Understanding how clinical boredom alters the stress response and modulates the reward system would possibly allow to identify precise molecular targets for pharmacological interventions (Clay et al. 2024), while also integrating genetic variables and the impact of low‐stimulus environmental contexts, the relevance of which has emerged forcefully during the pandemic emergency (Bambrah et al. 2023). Finally, the lack of controlled clinical studies that consider boredom reduction as a primary outcome highlights the urgent need to validate standardized intervention protocols through an interdisciplinary approach that integrates the contributions of psychology, psychiatry, and neuroscience. Only a systematic assessment of boredom in rehabilitation programs will allow it to be converted into a fundamental therapeutic lever for restoring cognitive engagement and existential meaning (Tagliaferri et al. 2025).

## 4 Conclusions

Current research indicates that boredom may represent a clinically relevant psychopathological dimension involving difficulties in attention regulation and meaning attribution, although its independence from related constructs remains uncertain. This condition was associated with a desynchronization between mental activity and the environment, rooted in structural and functional alterations in salience and reward circuits preventing the transition to rewarding cognitive engagement. The intrinsically transversal nature of the phenomenon supports its investigation as a possible heterogeneous nosological categories under a common attentional‐motivational denominator. Boredom has been associated with quality of life and response to treatment, possibly serving as a powerful risk factor for chronicity and the onset of adverse outcomes. Consequently, adopting a transdiagnostic perspective requires clinical interventions aimed at rehabilitating agency and the connection between the individual and their internal and external reality. Integrating this assessment into diagnostic and therapeutic protocols might enrich the paradigms of contemporary and inform interventions aimed at improving agency and engagement. Longitudinal, mechanistic, and intervention studies are needed to clarify its causal, prognostic, and therapeutic relevance.

## Author Contributions

Conceptualization: R.G., D.M. Methodology: R.G., D.M., M.G., G.S., E.P., F.M. Formal analysis: R.G., D.M. Data curation: R.G., D.M. Investigation: R.G., D.M., M.G., G.S., E.P., F.M. Visualization: R.G., D.M. Writing – original draft: R.G., D.M., M.G., G.S., E.P., F.M. Writing – review and editing: R.G., D.M. Supervision: D.M. Project administration: D.M.

## Funding

Open access publishing facilitated by Scuola Superiore Sant'Anna, as part of the Wiley ‐ CRUI‐CARE agreement.

## Ethics Statement

Ethical approval and informed consent were not required because this narrative review did not involve human participants, animals, or the collection of primary data.

## Conflicts of Interest

The authors declare no conflicts of interest.

## Data Availability

No new data were created or analyzed in this study. Data sharing is therefore not applicable to this article.
